# Forecasting the Population-Level Impact of Reductions in HIV Antiretroviral Therapy in Papua New Guinea

**DOI:** 10.1155/2011/891593

**Published:** 2010-12-01

**Authors:** Richard T. Gray, Lei Zhang, Tony Lupiwa, David P. Wilson

**Affiliations:** ^1^National Centre in HIV Epidemiology and Clinical Research, Faculty of Medicine, The University of New South Wales, Ground floor, CFI Building, Corner Boundary & West Streets, Darlinghurst, NSW 2010, Australia; ^2^National AIDS Council Secretariat, Waigani Drive, Boroko, NCD, Papua New Guinea

## Abstract

Papua New Guinea (PNG) recently did not secure external funding for the continuation of its antiretroviral treatment (ART) programs meaning that supplies of HIV drugs for the estimated 38,000 people living with HIV in PNG could be completely depleted during 2010. Using a mathematical model of HIV transmission calibrated to available HIV epidemiology data from PNG, we evaluated the expected population-level impact of reductions in ART availability. If the number of people on ART falls to 10% of its current level, then there could be an approximately doubling in annual incidence and an additional 12,848 AIDS-related deaths (100.7% increase) over the next 5 years; if ART provision is halved, then annual incidence would increase by ~68%, and there would be an additional ~10,936 AIDS-related deaths (85.7% increase). These results highlight that maintenance of ART and associated services through external funding is essential for the health and well-being of HIV-positive people in PNG.

## 1. Introduction

Papua New Guinea (PNG) is a low income country that has experienced a rapidly expanding HIV epidemic [1–3]. It has the highest HIV prevalence and incidence rate in the Pacific region [1] with 28,294 HIV infections reported by December 2008 since the first diagnosed case in 1987 [2]. The vast majority of HIV cases have been due to heterosexual transmission with similar numbers of diagnoses in men and women [2, 3]. Fortunately, recent estimates suggest there has been a leveling out of HIV prevalence in PNG at approximately 1% [4]. The reasons for this leveling of prevalence are currently unknown but could be due to the saturation of HIV in particular at risk population groups or geographic areas, or reflect the impact of the roll-out of intervention programs in recent years and the successful scale-up of antiretroviral therapy (ART) services across the country. ART first became available in PNG in 2004 and the PNG National Department of Health recently estimated that more than 70% of people requiring treatment were receiving it in 2009 [2, 4].

Despite experiencing high levels of economic growth, mainly from the natural resources sector, PNG has limited resources available for its HIV programs and treatment services [5, 6]. In addition, the distribution of these programs and services is limited by a lack of infrastructure and transport facilities due to PNG's mountainous and rugged terrain; over 85% of the population live in rural areas with only 3% of roads paved and many villages can only be accessed by foot [6]. Almost all of its resources for HIV services in PNG rely on external sources and in early 2010 PNG did not secure funding from the Global Fund for the continuation of its ART programs. This could have resulted in the complete depletion of ART. Fortunately, the PNG government provided resources to cover the costs of PNG's ART programs until the next round of funding in 2011 but has not been able to continue expansion of ART rollout. Without this government intervention, which impacts on an already limited and constrained health budget, the availability of life-sustaining ART would likely have had a large impact on the health and well-being of HIV-infected people in PNG and potentially result in a worsening epidemic. 

To investigate the potential population-level impact of a rapid fall in ART supplies in PNG, we developed a mathematical model to describe the HIV epidemic in PNG incorporating country specific demographic, epidemiological, behavioral, and clinical data. This model was calibrated to accurately reflect the estimated HIV incidence and prevalence, the number of recorded HIV diagnoses, and the number of people on ART in PNG. The model was then used to forecast the epidemic trajectory and number of AIDS deaths over the next 5 years under scenarios that the number of people receiving ART: (i) continues to increase following current trends, (ii) is maintained at the 2009 number, (iii) decreases by 50%, and (iv) decreases by 90%. We also investigated scenarios where there was a decrease by 50% and 90% for a two-year period before the re-establishment of the current roll–out; this coincides with the next round of possible funding from the Global Fund. Considering the limited antiretroviral stockpiles in PNG and reliance on foreign funds, scenarios involving substantial declines in ART access are not unrealistic.

## 2. Materials and Methods

A mathematical HIV transmission model was developed to describe the history of the HIV epidemic in PNG and to forecast potential epidemic trends in the future due to reductions in ART availability. All model simulations were executed with Matlab R2009a.

### 2.1. Model Specifications

**Figure 1:**
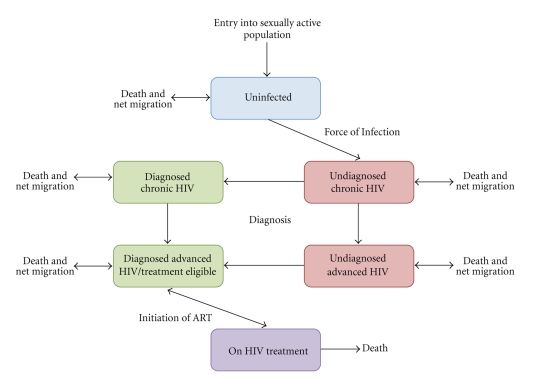
Schematic diagram of the stages of HIV infection described by the model and the progression of people through these stages due to infection, disease progression, diagnosis, and initiation of treatment. These stages are replicated for the urban and rural population in PNG with migration (except for people on treatment) between corresponding infection stages. People enter the uninfected population when they become sexually active and leave the population due to natural or HIV-related mortality.

The HIV transmission model describes the movement between twelve population compartments within both urban and rural sexually active populations. Urban HIV susceptible individuals (*S*_*u*_) may become infected based on probabilistic rates that inform a deterministic “force of infection”. Newly infected individuals enter into the undiagnosed chronic/asymptomatic stage (*C*_*u*_^*u*^). Chronically infected individuals can progress to advanced HIV/ AIDS (*A*_*u*_^*u*^) and people in chronic or advanced HIV stage can become diagnosed with their infection (*C*_*u*_^*d*^, *A*_*u*_^*d*^). Only diagnosed advanced HIV patients can begin ART (*T*_*u*_). The same model applies to the rural population with similar disease stages, denoted by *S*_*r*_, *C*_*r*_^*u*^,*C*_*r*_^*d*^, *A*_*r*_^*u*^, *A*_*r*_^*d*^, and *T*_*r*_.  A schematic diagram of the model is shown in Figure 1. In the model, individuals can migrate between urban and rural settings while remaining in the same disease stage and sexual mixing occurs among people in the same (urban or rural) setting. It is assumed that ART patients remain at their current location for treatment services. 

The overall population-level transmission and disease progression of HIV is described by the following set of equations:

(1) $$\begin{array}{cc} \frac{dS_{x}}{dt} & =\pi_{x}-\left(\Lambda_{x}+\mu +\nu_{x}\right)S_{x}+\nu_{y}S_{y},\\ \frac{dC_{x}^{u}}{dt} & =\Lambda_{x}S_{x}-\left(\gamma +\delta_{x}^{c}+\;\mu_{c}+\nu_{x}\right)C_{x}^{u}+\nu_{y}C_{y}^{u},\\ \frac{dC_{x}^{d}}{dt} & =\delta_{x}^{c\;}C_{x}^{u}-\left(\gamma +\mu_{c}+\nu_{x}\right)C_{x}^{d}+\nu_{y}C_{y}^{d},\\ \frac{dA_{x}^{u}}{dt} & =\gamma C_{x}^{u}-\left(\mu_{a}+\delta_{x}^{a}+\nu_{x}\right)A_{x}^{u}+\;\nu_{y}A_{y}^{u},\\ \frac{dA_{x}^{d}}{dt} & =\delta_{x}^{a}A_{x}^{u}+\gamma C_{x}^{d}+\omega T_{x}-\left(\tau_{x}+\mu_{a}+\nu_{x}\right)A_{x}^{d}+\;\nu_{y}A_{y}^{d},\\ \frac{dT_{x}}{dt} & =\tau_{x}A_{x}^{d}-\left(\omega +\mu_{t}\right)T_{x}, \end{array}$$

where *π*_*x*_ and Λ_*x*_ denote the entry rate into the population and force of infection, respectively,

(2) $$\begin{array}{cc} \pi_{x} & =\rho \left(S_{x}+C_{x}^{u}+C_{x}^{d}+A_{x}^{u}+A_{x}^{d}+T_{x}\right), \end{array}$$

(3) $$\begin{array}{cc} \Lambda_{x} & =\frac{\beta \left(C_{x}^{u}+\;C_{x}^{d}+\theta_{a}\left(A_{x}^{u}+A_{x}^{d}\right)+\theta_{t}T_{x}\right)}{S_{x}+C_{x}^{u}+C_{x}^{d}+A_{x}^{u}+A_{x}^{d}+T_{x}}, \end{array}$$

and the subscripts *x*, *y* = *u*, *r*  denote urban and rural populations. The definition of each parameter in these equations is given in Table 1. 

To evaluate the impact of HIV treatment on transmission and the number of advanced HIV-related deaths, we combine sexual behavior and HIV transmission into a single parameter *β* that represents the rate of HIV infection for susceptible individuals with chronically infected partners. Due to a lack of data distinguishing sexual behavior in urban and rural areas, we assume the same value of *β* for urban and rural areas. Multiplicative factors *θ*_*a*_ and *θ*_*t*_ are used to take into account the increased infectiousness of advanced HIV patients and the decreased infectiousness of people on ART, as described in (3). We assume identical population growth rates, HIV-related death rates, rates of disease progression to advanced HIV, and ART drop-out rate for both urban and rural areas. The values used for these rates are based on the best available international evidence for comparable settings and are presented in Table 1. 

Model parameter values associated with ART are important in this analysis. Specifically, it was assumed that ART reduces the infectiousness of HIV-infected people by 92%. This is based on a recent meta-analysis [9] and a prospective cohort study [10] which each calculated a 92% reduction in HIV transmission rates from heterosexual men and women who are treated compared with those who are untreated. We also assumed that in PNG 5% of people on ART die per year, compared with 30% of people with advanced HIV disease [11, 12].

### 2.2. Model Fitting and Calibration

The epidemic model is calibrated against available data on HIV prevalence, HIV incidence, the number of HIV diagnoses, and the number of people on ART for PNG from 1990 to 2008 [2, 4]. To fit data, the values of *β*, *δ*_*u*_^*c*^, *δ*_*u*_^*a*^, *δ*_*r*_^*c*^, *δ*_*r*_^*a*^, *τ*_*u*_, and *τ*_*r*_ were varied over time while the other parameters remained constant (Table 1). The population growth rate *ρ* and the urban-rural migration rates *ν*_*u*_ and *ν*_*r*_ were fixed for 1990–2008 to match World Bank estimates and PNG Census data [13, 15]. 

The overall force of infection for HIV in PNG (Λ) was estimated by adding the change in the number of HIV-infected patients to the estimated number of HIV-related deaths and dividing by the overall size of susceptible population (3). The fitted force of infection was then used to determine the value of *β* using (3) and the fact that Λ(*S*_*u*_ + *S*_*r*_) = Λ_*u*_ *S*_*u*_ + Λ_*r*_ *S*_*r*_. The resulting fitted value of *β* has a value of 0.256 at the start of 1990, reaches a peak value of 0.443 just prior to 2000, and then rapidly falls to a minimum value of 0.064 by the end of 2008. The parameter *β* refers to the probability of transmission occurring per year in a long-term discordant partnership in which the HIV-infected person is in the chronic stage; a range of 0.05–0.25 was reported among different stratifications of discordant partners in an African settings suggesting that our estimates are not unrealistic [7].

The only diagnoses data available was the annual diagnoses for all of PNG. We assumed values for the proportion of diagnoses that occur in urban areas *ϕ*_*u*_ and the proportion of diagnoses that occur in advanced HIV patients *ϕ*_*a*_. Until 1996 the only location for HIV testing in PNG was at the Port Moresby General Hospital [3]. Since then, numerous testing sites have been established across the country, but urban residents still have more accessibility to HIV testing facilities. This suggests that there should be no diagnoses of HIV in rural areas until 1996; however, expert opinion and anecdotal evidence suggests that many people travelled to Port Moresby for HIV testing. Therefore, for fitting and simulation purposes, we assume 20% of HIV/AIDS infected patients are from urban areas (slightly higher than the proportion living in urban areas). In addition, we assume that 75% of HIV diagnoses occur when an infected person has developed advanced HIV symptoms. These assumptions result in diagnoses rates that slowly increase until 2003 before rapidly increasing until the end of 2008, matching the actual rapid expansion in HIV testing across PNG in recent years [3]. The model-fitted diagnoses rates were generally 2-3 times higher in urban areas and 2–4 times higher in the advanced HIV population groups, which are consistent with the perceived situation in the country. The fitted testing parameters values at the end of 2008 correspond to an undiagnosed advanced HIV patient taking on average from 5 to 16 months to be diagnosed in urban and rural areas, respectively. 

Rates at which diagnosed chronic patients go onto treatment (*τ*_*u*_, *τ*_*r*_) are calculated based on available data on the number of people starting ART in PNG since 2004 when ART first became available. As there are no available data for the location of residence for patients on ART, we assumed that the rate at which diagnosed AIDS patients initiate ART in urban areas is twice that in rural areas. The rates of initiating treatment rapidly increase from zero in 2004 and match the roll-out of ART services that cover an estimated 80% of treatment-eligible people in 2009 [2, 4].

**Figure 2:**
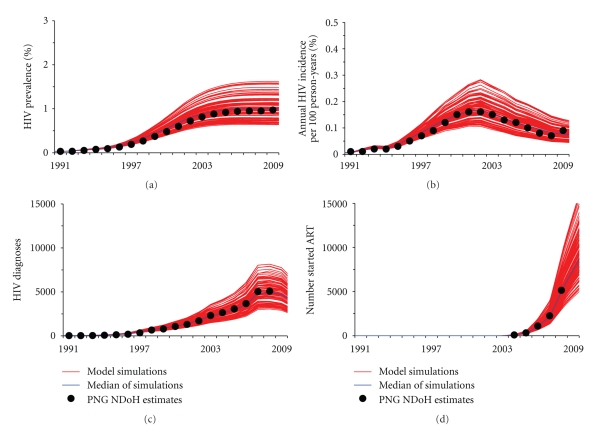
Fitting and calibration of the HIV transmission model to PNG epidemiological data from 1990 to 2009. In each figure, estimates or data from the PNG National Department of Health (black discs, data) are compared with 100 model-based simulations (red) and median simulations (blue) over the period from 1990 to 2009. (a) HIV prevalence in adult population. (b) HIV incidence each year per 100 persons. (c) Number of HIV diagnoses each year. (d) The cumulative number of HIV-infected people who have started antiretroviral treatment by the end of each year.

Due to gaps in data and the absence of an effective and systematic reporting system, there are large uncertainties surrounding HIV epidemiological data in PNG. To incorporate these uncertainties, we assume a range of ±10% for each of the fitted parameter values (see Table 1). One hundred parameter sets were used in the analysis, sampled (through Latin hypercube sampling implemented in the SaSAT computer program [16]) from the parameter ranges specified in Table 1 (Figure 2).

### 2.3. ART Reduction Scenarios

To understand the future impact of ART reductions in PNG, we considered four scenarios. These were simulated by our model for the next five years for each of our parameter sets. These scenarios are specified by the number of people on ART. The scenarios are as follows:

- continual roll-out of ART which extend current trends in PNG. This is the baseline scenario used for comparison with other scenarios and is simulated by extending the period of simulation with the parameters fixed to their values at the end of 2009,
- fixing the number of people on ART to a constant value as at the end of 2009. This represents a consistent supply of ART in PNG that sustains a fixed number of people on ART into the future,
- reducing the number of HIV-infected people on ART by 50% by 2015,
- reducing the number of HIV-infected people on ART by 90% by 2015.

**Figure 3:**
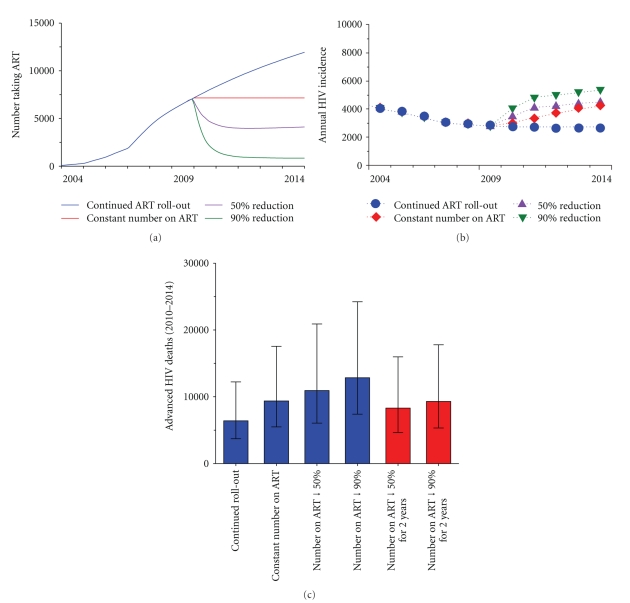
The impact of reductions in ART availability. (a) Median number of HIV-infected people receiving ART for scenarios from (i) to (iv) in the text. These scenarios were a continuation of the ART roll-out after 2009, maintaining ART services so that the number of people who receive ART remains constant, a reduction in the number of people receiving treatment of 50% from the level at the end of 2009, and a reduction in the number of people receiving treatment of 90% from the level at the end of 2009. (b) Median annual incidence per 100 person-years after 2009 for 100 simulations of each of the scenarios considered. (c) Total number of advanced HIV deaths for the period 2010–2014 for all the scenarios considered. The blue bars represent the median number of deaths for scenarios from (i) to (iv) in the text with the black error bars showing the minimum and maximum number of deaths for 100 model simulations of each scenario. Similarly, the red bars represent the median number of deaths for the scenarios where ART programs and services are interrupted for two years.

The variation in number of people on ART from the end of 2009 for each of these scenarios is shown in Figure 3(a). We also investigated two scenarios where the reduction in ART services only lasted for two years before the re-establishment of the current ART roll-out (potentially due to a successful funding application in the next Global Fund round in 2011). In these two scenarios, the parameter values were set to the values in scenario (iii) and (iv), respectively, before returning to their current values after two years. The number of people on ART follows the same trend as the (iii) and (iv) scenarios for two years before rapidly increasing towards the expected number under current conditions.

## 3. Results and Discussion

Using our mathematical model we evaluated the impact of reductions in ART roll-out in PNG by forecasting the change in expected HIV incidence and advanced HIV deaths in PNG over the 5 years for each scenario. Our results show that a reduction in ART availability in PNG is likely to have a large and significant impact on the HIV epidemic. 

### 3.1. Increase of Annual HIV Incidence

Our model predicts that a continual roll-out of ART in PNG would result in a declining trend in HIV incidence. The annual HIV incidence is predicted to reduce from a median of 2,794 infections (0.074 per 100 person-years, IQR 2,227–3,360) in 2009 to 2,674 infections in 2014 (0.062 per 100 person-years, IQR 2,109–3,239) (Figure 3(b)). If the continual roll-out of ART is interrupted in 2010, then a large rise in annual HIV incidence is expected (Figure 3(b)). For the scenario where the number of ART patients remains at a constant level after 2009, the expected number of new HIV infections in 2014 will be 4,253 (0.099 per 100 person-years, IQR 3,264–5,242) which is a 59% increase from the baseline level. If the number of people on ART is reduced by 50% or 90%, then incidence is forecasted to increase to 4,490 (0.10% per 100 person-years, IQR 3,382–5,599) and 5,323 (0.12% per 100 person-years, IQR 4,035–6,610) in 2014, which, correspond to 67.9%, and 99.1% increases, respectively. Cumulatively, for scenarios (ii), (iii), and (iv), 4,871, 7,181, and 10,934 additional HIV cases (compared to baseline) are estimated to occur during 2010–2014 for these scenarios. Further, if ART availability remains low beyond 2015, higher incidence levels will likely result. If the 50% and 90% reductions in ART are only in effect for two years, there is still a substantial increase in the expected incidence over the two-year period before declining to a level slightly above forecasts for current conditions. According to these two scenarios, it could be expected that ~3,298 and 5,342 additional HIV cases (compared to baseline) will occur during 2010–2014 period, respectively.

This increase in incidence is likely to be an upper bound as the sexual behavior and prevalence of other sexually transmitted infections (STIs) affecting HIV transmission are assumed to be constant in our model; however, these factors are implicitly incorporated in our epidemiological fitting routine. In reality, sexual behavior could change due to the continuation of other education and prevention programs or because people are sick with advanced HIV. Furthermore, the level of STIs in the population is expected to change with the expansion of voluntary counseling and testing services and targeted treatment programs. On the other hand, many of these programs could also be affected by decreases in HIV funding potentially leading to larger increases in incidence. By only focusing on ART, our results show the relative impact of reductions in ART availability on the PNG HIV epidemic. The effect of other factors that could also change requires separate evaluation; however, the direction and impact of decreases in funding on these factors are unknown.

### 3.2. Increase of Advanced HIV-Related Deaths

With the continuation of the current ART roll-out, the total number of advanced HIV deaths in PNG over 2010–2014 is forecasted to be 6,402, (range 3,714–12,221) which corresponds to an average annual death rate of 1.52% in the population of people living with HIV. Figure 3(c) highlights the severe increase in the number of advanced HIV deaths resulting from a reduction in ART availability. Our model predicts an additional 2,975 (range 1,783–5,333) advanced HIV deaths between the beginning of 2010 and the end of 2014 if the supply of ART is maintained at the current level. This corresponds to a 46.5% increase in the number of advanced HIV deaths in comparison with the continuous ART roll-out scenario. Further, for the scenarios where the number of patients on ART reduces to 50% and 10% of the 2009 level, the median number of cumulative deaths over the period 2010–2014 will increase to 10,936 (range 6,047–20,891) and 12,848 (range 7,392–24,232), which correspond to 70.8% and 100.7% rise, respectively. This lifts the advanced HIV death rates of HIV-infected individuals to 2.5% and 2.9% per year. When ART supplies are only interrupted for two years the median number of cumulative deaths over the period 2010–2014 is projected to increase to 8,300 (range 4,651–15,969) and 9,299 (range 5,323–17,786) for the 50% reduction and 90% reduction scenarios, respectively. A large reduction over this short period results in almost as many deaths as maintaining the supply of ART at the current level (Figure 3(c)).

## 4. Conclusions

The HIV epidemic in PNG has been in a large expansion phase over the past 15 years. Although the prevalence of HIV has likely started to reach a plateau, prevention and control measures require renewed efforts. Thus far, the epidemic has resulted in an estimated 38,000 people becoming infected with HIV in PNG with over 28,000 of these diagnosed. To remain healthy, these people require clinical care and management of their infection and access to effective combination antiretroviral therapy. As a lower income country, PNG has not been able to finance the required antiretroviral drugs for its citizens and has depended on external funding for its ART programs. Unfortunately, PNG recently did not secure external funding for the continuation of these vital programs and the Papua New Guinean government had to allocate funding from already constrained health budgets to continue the roll-out of ART. Without funding, ART availability in PNG would not increase according to scale-up plans and could decrease very substantially. 

In this study, we used a mathematical model to demonstrate that if ART supplies are not funded and declined, then substantial population-level impacts could be expected in PNG. Specifically, if the number of people on ART falls to 10% of current level, then over the next 5 years there could be an approximate doubling in annual incidence and an additional 12,848 AIDS-related deaths. Even if ART provision is halved then annual incidence would increase by ~68% and there would be an additional ~10,936 AIDS-related deaths. 

The model we developed was calibrated to accurately reflect the unique epidemiology of PNG at the overall population-level and was based on the best data available; however, it is a relatively simple model and could not capture the full degree of complexity and heterogeneity that exists within the population. In particular, the model did not capture the movement of HIV-positive people accurately, did not investigate the impact on particular at risk population groups such as commercial sex workers, and did not consider treatment failure and the possible increased emergence of drug-resistant strains of HIV. Regardless, the model is useful for obtaining the population-level relative change in new incident cases and AIDS-related deaths that could result due to a drug shortage in PNG. These results highlight the large degree of crisis for people at risk of or living with HIV in PNG and their families and community. This crisis also affects other concerning health issues such as tuberculosis; the HIV prevalence in tested tuberculosis patients is ~3.5% in PNG [2]. A similar population-level relative impact could be expected for other low and middle income settings that rely on external funding for ART supplies if their ART funding were to reduce significantly.

The majority of funds for HIV prevention, care, and treatment in PNG are externally funded from the Global Fund, international government development organizations, and nongovernment organizations. While PNG's economy has experienced relatively strong growth, many international organizations suffered a reduction in funding after the 2008 global financial crisis which could lead to impacts on HIV/AIDS epidemics [17, 18]. A judicious mix of funding sources and disbursement channels could be important for responding to HIV epidemics in PNG [19]. This includes the PNG government which may need to consider taking greater responsibility for funding HIV programs to reduce exposure of these programs to external volatility, especially since Government expenditure for HIV has decreased in recent years. Irrespective of funding sources, it must be acknowledged that there could be a state of emergency for people living with HIV in PNG. People with late-stage HIV infections do not have access to effective ART and are at real risk of premature death. Therefore, it is of very high importance that funding for HIV programs are obtained and that appropriate administration processes are put in place to handle any funds procured and manage the logistics of channeling ART for effective distribution. In addition, improvements in infrastructure are required to reach and care for people with HIV in PNG. These complex factors need to be addressed in PNG and in other similar low-resource settings in the process of reaching a major goal of achieving universal treatment access for all people living with HIV.

## Figures and Tables

**Table 1:** Definitions and value ranges for input and fitting parameters used in our HIV transmission model.

Parameter	Description	Value (Range)	Footnote/Reference
*Demographic parameters*			

*N*_0_	Initial size of sexually active population	2,317,648	(a)
*ρ*	Population growth rate	4.8% (4.7–4.9%)	

*α*_0_	Initial proportion of population in urban areas	15%	(b)
*ν*_*u*_	Migration rate from urban to rural settings	2.25% (2–2.5%)	
*ν*_*r*_	Migration rate from rural to urban settings	0.2% (0.18–0.23%)	

1/*μ*	Average duration that a person remains in the sexually active population	45 years	Assumed

*HIV Biological parameters*			

*β*	Rate at which susceptible individuals with partners that are chronically infected become infected with HIV	fitted (±10%)	(c)

*θ*_*a*_	Multiplicative increase in HIV transmission probability for partners with AIDS	5 (4–6)	[7, 8]
*θ*_*t*_	Multiplicative decrease in HIV transmission probability for HIV-infected partners on effective ART	0.08 (0.05–0.13)	[9, 10]
*γ*	Disease progression rate at which individuals chronically infected with HIV progress to AIDS	0.075 (0.06–0.09)	[7, 9]
*μ*_*c*_	Death rate per year for a person chronically infected with HIV	3%	[11, 12]
*μ*_*a*_	Death rate per year for individuals with AIDS	30%	[11, 12]
*μ*_*t*_	Death rate per year for HIV-infected individuals on ART	5%	[11, 12]

*Clinical parameters*			

*δ*_*u*_^*c*^	Diagnosis rate for urban individuals with chronic infection	fitted (±10%)	(e)
*δ*_*u*_^*a*^	Diagnosis rate for urban individuals with AIDS	fitted (±10%)	
*δ*_*r*_^*c*^	Diagnosis rate for rural individuals with chronic infection	fitted (±10%)	
*δ*_*r*_^*a*^	Diagnosis rate for rural individuals with AIDS	fitted (±10%)	

*τ*_*u*_	Rate at which urban individuals diagnosed with AIDS begin treatment	fitted (±10%)	(f)
*τ*_*r*_	Rate at which rural individuals diagnosed with AIDS begin treatment	fitted (±10%)	

1/*ω*	Average duration at which individuals remain on ART	10 years (6.7–20 years)	(g)

^(a)^To calibrate the model to the HIV epidemic in PNG from 1990 to 2010, the sexually active population size was assumed to be equal to the 15–49-year-old adult population estimated by the World Bank [13]. From the initial 1990 population, the value of *ρ* (which is assumed to be the same for urban and rural areas) is set to match the growth of the 15–49-year-old population seen in PNG from 1990 to 2008 [13].

^(b)^Data from the World Bank gives the proportion of the population that is living in urban areas to be approximately 15%. However, the proportion of the population in urban areas has been declining very slightly from 1990 to 2008 [13]. The migration rates between urban and rural areas are set to follow this trend after the urban proportion is set to be initially 15%.

^(c)^This rate incorporates the number of sexual partners, condom usage, effect of STIs, and other factors that affect HIV transmission. As we are focused on the impact of ART reductions, we group these factors into a single rate which is then fitted to the annual prevalence and incidence estimates for PNG. The value of *β* is likely to be different for urban and rural areas; however, there is limited data available for these population categories so we have assumed the same value for fitting purposes.

^(d)^The value and range for *γ* have been used to match the estimates for the number of people who require ART in PNG. The disease progression rate we use corresponds to an average duration of being chronically infected equal to 11–16.6 years which is much longer than established durations for low resource settings [14]. Due to the simplicity of our HIV transmission model, which only has one infection stage prior to AIDS, a longer average time period is required to match the actual epidemiology.

^(e)^The rate at which infected people are diagnosed is fitted to match the overall number of diagnoses recorded in PNG [2].

^(f)^The rate at which individuals diagnosed with AIDS begin treatment is fitted to match the overall number of people who have started treatment since 2003, the estimated number of people who require treatment, and the ART coverage in PNG [2, 4].

^(g)^The average duration at which urban and rural people remain on ART is likely to be different due to supply and logistical issues. However, there is very little data on the length of time people remain on ART in PNG; hence, we have assumed the same value and range for urban and rural areas.
